# Nerve sprouting capacity in a pharmacologically induced mouse model of spinal muscular atrophy

**DOI:** 10.1038/s41598-019-44222-2

**Published:** 2019-05-24

**Authors:** Mendell Rimer, Bonnie L. Seaberg, Pei-Fen Yen, Steven Lam, Robert Louis Hastings, Young il Lee, Wesley J. Thompson, Zhihua Feng, Friedrich Metzger, Sergey Paushkin, Chien-Ping Ko

**Affiliations:** 1 Department of Neuroscience and Experimental Therapeutics Texas A & M University Health Science Center, Bryan, TX USA; 2 Section of Neurobiology Department of Biological Sciences University of Southern California, Los Angeles, CA USA; 3 Department of Biology Texas A & M University, College Station, TX USA; 4 Roche Pharmaceutical Research and Early Development, Roche Innovation Center Basel, Basel, Switzerland; 5 SMA Foundation, New York, NY USA

**Keywords:** Developmental disorders, Motor neuron disease

## Abstract

Spinal muscular atrophy (SMA) is caused by loss-of-function mutations in the survival of motoneuron gene 1 (*SMN1*). SMA is characterized by motoneuron death, skeletal muscle denervation and atrophy. Disease severity inversely correlates with copy number of a second gene (*SMN2*), which harbors a splicing defect that causes the production of inadequate levels of functional SMN protein. Small molecules that modify *SMN2* splicing towards increased production of functional SMN significantly ameliorate SMA phenotypes in mouse models of severe SMA. At suboptimal doses, splicing modifiers, such as SMN-C1, have served to generate mice that model milder SMA, referred to as pharmacological SMA mice, which survive into early adulthood. Nerve sprouting at endplates, known as terminal sprouting, is key to normal muscle fiber reinnervation following nerve injury and its promotion might mitigate neuromuscular symptoms in mild SMA. Sprouting has been difficult to study in severe SMA mice due to their short lifespan. Here, we show that pharmacological SMA mice are capable of terminal sprouting following reinnervation that is largely SMN-C1 dose-independent, but that they display a reinnervation delay that is critically SMN-C1 dose-dependent. Data also suggest that SMN-C1 can induce by itself a limited terminal sprouting response in SMA and wild-type normally-innervated endplates.

## Introduction

Spinal muscular atrophy (SMA) is an autosomal recessive neuromuscular disease characterized by motoneuron loss, muscle denervation and atrophy, caused by a scarcity of full length survival of motoneuron (SMN) protein^1^. SMA is a major genetic cause of infant mortality^2^. The gene mutated in SMA is *SMN1*^3^. In humans, a second, almost identical gene, *SMN2*, produces low levels of full-length SMN protein, but most of its transcripts lack exon 7 and encode an unstable, minimally functional protein (SMNΔ7)^4,5^. Severity of SMA is inversely related to *SMN2* copy number^6^.

Mice only carry one SMN gene, whose homozygous deletion (*Smn*^−/−^) is embryonic lethal. *Smn*^−/−^ mice with two copies of *SMN2* (i.e. *Smn*^−/−^*; SMN2*^+/+^) die perinatally and exhibit features resembling the most severe human disease^7^. Addition of a transgene encoding the cDNA for SMN∆7 to these mice (i.e. *Smn*^−/−^*; SMN2*^+/+^*; SMN∆7*) improves survival to 14 days on average. These animals, known as SMN∆7 mice, have been widely used in the field to model severe forms of SMA, which has the highest incidence rate in the population^8^. A pharmacologically-induced SMA mouse model was developed^9^ by treating SMNΔ7 mice with a suboptimal dose of a *SMN2* splicing modifier (SMN-C1)^10^, which increases the levels of full-length SMN produced from *SMN2* by promoting exon 7 inclusion into mature transcripts. These mice have milder SMA phenotypes than SMNΔ7 mice and survive into adulthood, allowing testing of SMA treatments after disease onset.

Motoneurons send out axons that synapse onto skeletal muscle fibers at the endplate (a.k.a. neuromuscular junction (NMJ))^11^. NMJs have three cellular components, the presynaptic axon terminal, the postsynaptic sarcolemma where acetylcholine receptors (AChRs) cluster, and terminal Schwann cells (tSCs), a.k.a. perisynaptic Schwann cells^12^, non-myelinating glia that cap the nerve terminal. Axons degenerate distally to a peripheral nerve injury, but later return to the original denervated endplates by following endoneurial tubes filled with Schwann cells^13^. Upon arriving at the synaptic site, axons follow cellular processes that extend beyond the endplate, which are generated by tSCs that remain at endplates during the denervation period^14–16^. Terminal sprouts are thus made up of axonal sprouts that grow on tSC sprouts. Terminal sprouts from an endplate can innervate nearby endplates that are not contacted by the main axonal branches. Hence, this compensatory nerve sprouting is important to normal muscle reinnervation following nerve injury in adults^17^, and its promotion might mitigate neuromuscular symptoms in mild SMA. While the capacity for nerve sprouting is preserved in *Smn*^+/−^ mice, mouse models for the mildest forms of SMA^18,19^, it has been difficult to study sprouting in SMNΔ7 mice due to their severe phenotype and short lifespan. Here, we tested whether nerve sprouting occurs after reinnervation in the pharmacological SMA model mice and how it is impacted by the dose of SMN-C1 treatment.

## Results

**Figure 1:**
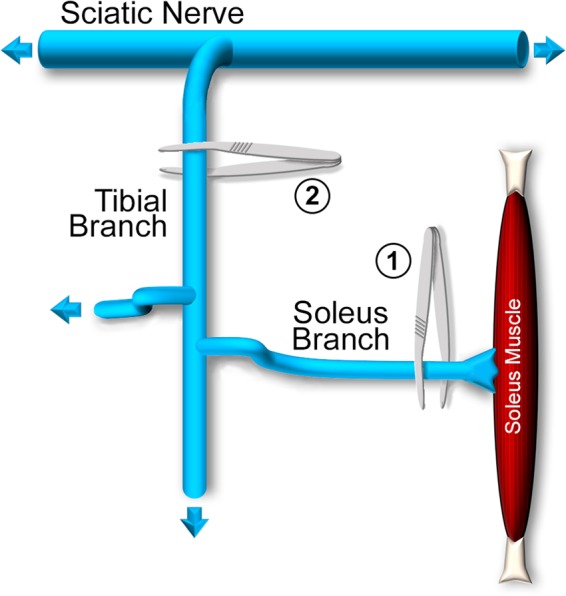
Schematic diagram of surgical denervation procedures. Two types of denervation experiments were performed; both allowed for reinnervation of muscle by the crushed axons. In 1, the soleus branch of the sciatic nerve was crushed with forceps close to its entry into the soleus muscle. In 2, the tibial branch of the nerve was crushed much farther away from the soleus muscle. Not drawn to scale.

We examined terminal sprouting capacity after nerve crush of the sciatic nerve branch that innervates the soleus muscle (SOL). Starting at postnatal day 1 (PND1), SMNΔ7 mice were given daily SMN-C1 IP injections at low (LD, 0.1 mg/kg) or high (HD, 3 mg/kg) doses. These dose regimes are, respectively, therapeutically suboptimal and optimal in SMAΔ7 mice^9,10^. Age- and strain-matched, wild type (WT) mice injected with DMSO vehicle were used as controls. Between PND31 and PND41, the right SOL was denervated by crushing the soleus branch of the tibial nerve at the site of muscle entry (Fig. 1, crush 1). After a 7-day period to allow reinnervation, animals were euthanized and SOL from denervated and contralateral legs were dissected and processed for whole-mount immunostaining of axons and nerve terminals, Schwann cells and AChRs that mark synaptic sites. Nerve terminal sprouting was assessed on confocal stacks and quantified by scoring three parameters: (i) endplates with sprouts (%); (ii) number of sprouts per endplate, and (iii) sprout length.

### Increased number of short nerve terminal sprouts in contralateral SMA muscles

**Figure 2:**
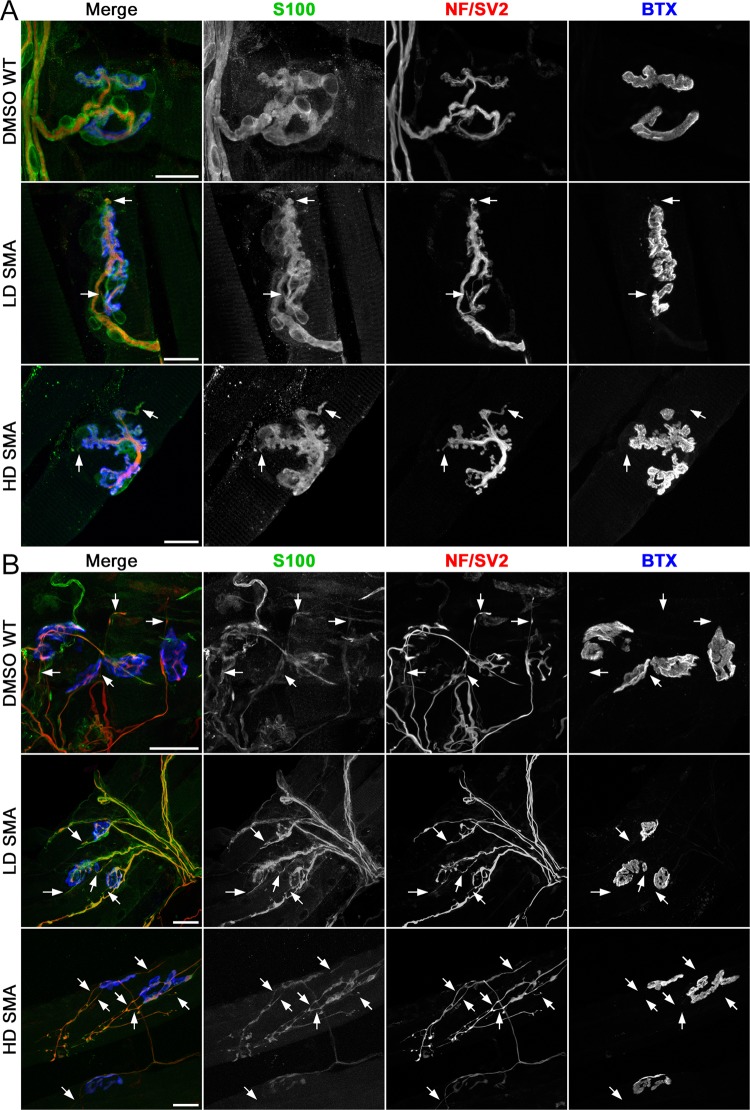
Terminal sprouting at endplates in contralateral and reinnervated SOL muscles. Confocal maximum projections of endplates after whole mount staining for Schwann cells (S100), axons and nerve terminals (NF/SV2) and AChRs (BTX). Arrows point to terminal sprouts. (**A**) Contralateral SOL: An example of a NMJ without terminal sprouts in DMSO WT SOL, and two examples of NMJs with two short terminal sprouts each in LD and HD SMA SOL. (**B**) Denervated SOL: Endplates with more profuse terminal sprouting 7 days after crush denervation in all treatment groups. Scale bars: 20 μm (**A**). 30 μm (**B**).

**Figure 3:**
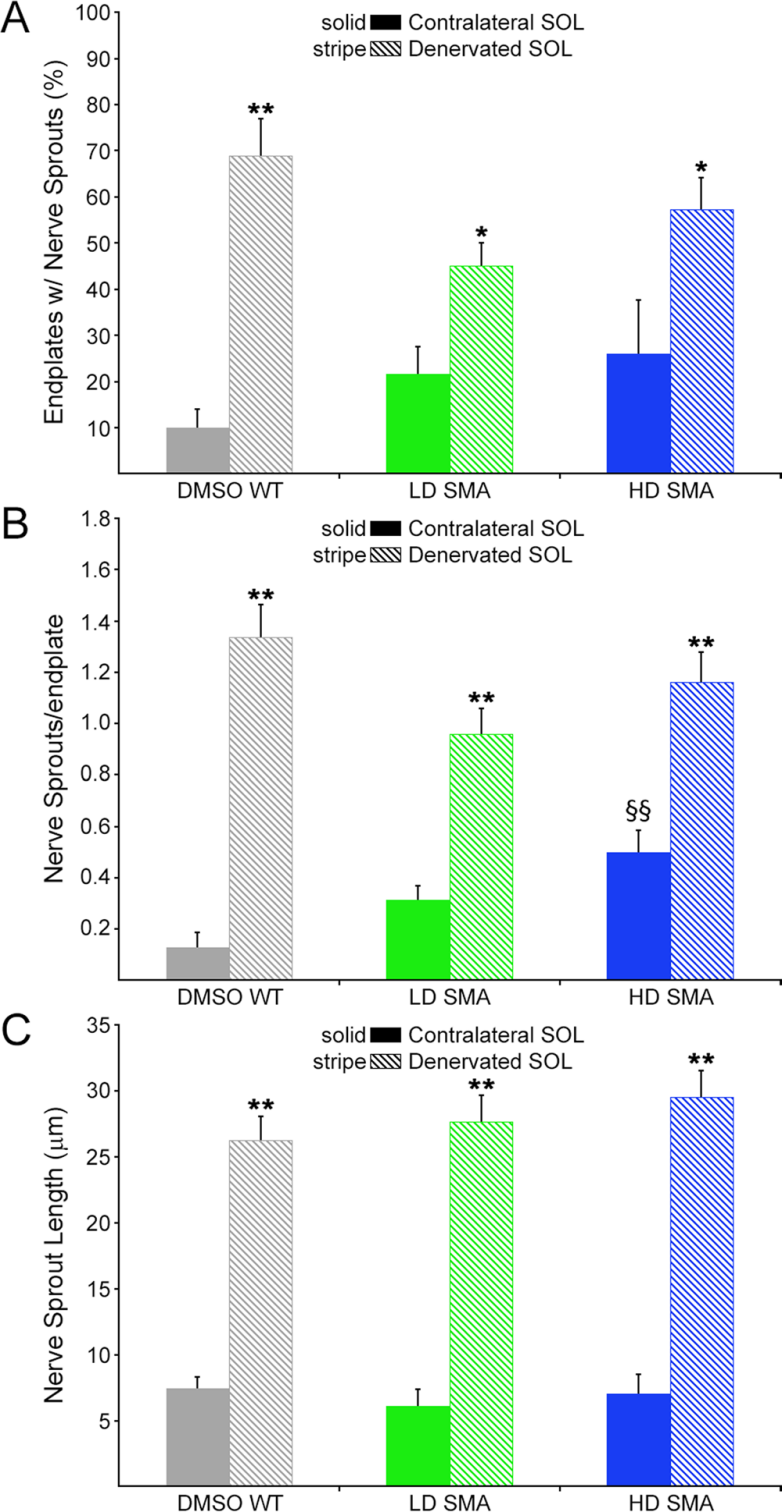
Reinnervation induced more and longer terminal sprouts in both DMSO WT and pharmacological SMA SOL. Quantification of terminal nerve sprouting in contralateral (solid bars) and denervated (striped bars) SOL. Percentage of endplates with sprouts **(A**) nerve sprouts per endplate (**B**) and nerve sprout length (**C**). Sample sizes: contralateral SOL, (**A,B**) DMSO WT: 6 mice, 78 endplates; LD SMA: 8 mice, 160 endplates; HD SMA: 5 mice, 122 endplates. Denervated SOL, (**A,B**) DMSO WT: 5 mice, 90 endplates; LD SMA: 5 mice, 152 endplates; HD SMA: 5 mice, 141 endplates. Sprouts measured in contralateral and denervated SOL, respectively (**C**) DMSO WT: 10 and 111; LD SMA: 50 and 131; HD SMA: 57 and 160. *p < 0.05; **p < 0.01, t-test vs. contralateral side, same treatment group; ^§§^p < 0.01, ANOVA with Bonferroni post hoc test performed on contralateral data (see text).

**Figure 4:**
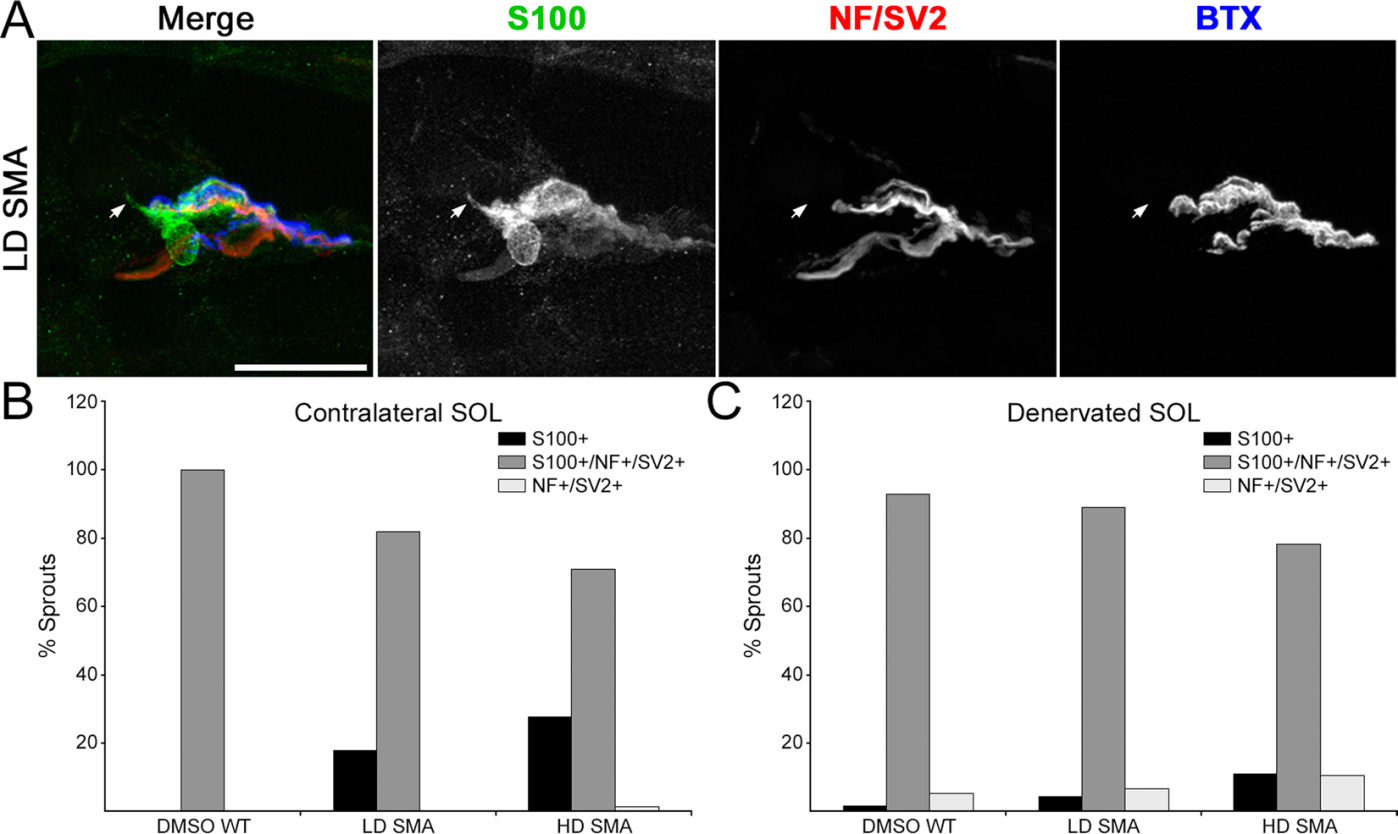
Cell marker distribution of terminal sprouts. (**A**) An example of a NMJ in a LD SMA contralateral SOL with one sprout (arrow) that labeled positively for S100 but negatively for NF/SV2. This was thus a tSC terminal sprout without an associated nerve sprout. Scale bar: 25 μm. **(B)** Cell marker distribution of terminal sprouts in contralateral SOL. While all sprouts labeled for both S100 and NF/SV2 in DMSO WT SOL, about 20% of sprouts only labeled for S100 in SMA muscles. **(C)** Cell marker distribution of terminal sprouts in reinnervated SOL. Most sprouts labeled for both S100 and NF/SV2 in all groups. Sprouts scored in contralateral and denervated SOL, respectively: DMSO WT: 10 and 114; LD SMA: 61 and 137; HD SMA: 79 and 180.

We first examined sprouting in contralateral (non-denervated) muscles to determine a baseline. Most NMJs in WT and SMA contralateral muscles had no terminal sprouts and showed the expected precise apposition between nerve terminal, tSC and AChR staining. However, in LD and HD SMA SOL, NMJs with short terminal sprouts (<15 μm-long) were readily found (Fig. 2A). Quantification showed that contralateral DMSO WT SOL averaged 10.05 ± 4.05% endplates with short nerve terminal sprouts, whereas LD SMA SOL averaged 20.78 ± 5.96% endplates with these sprouts and HD SMA SOL had 25.95 ± 11.62% endplates with such sprouts. (Fig. 3A, solid bars). The upward trend in NMJs with short nerve sprouts in contralateral SMA SOL relative to WT SOL was not statistically significant by ANOVA. The number of sprouts/endplate was also increased in both contralateral LD and HD SMA SOL and particularly, the number in contralateral HD SMA SOL (0.50 ± 0.08) was statistically higher than in contralateral DMSO WT SOL (0.13 ± 0.06; ANOVA: F (df 2, 357) = 5.793; p = 0.0033; t value DMSO WT vs HD SMA = 3.363) (Fig. 3B, solid bars). While 100% of the sprouts on the contralateral DMSO WT SOL stained positive for both Schwann cell (S100) and axon/nerve terminal markers (neurofilament (NF) and SV2, respectively), 18–28% of the sprouts on the LD or HD SMA SOL were positive only for S100 but not for NF/SV2, indicating that these were tSC sprouts that lacked axonal sprouts (Fig. 4A,B). To distinguish whether sprouts on endplates in contralateral SMA SOL resulted from the underlying disease process or from the SMN-C1 treatment, WT mice (n = 4) were given a daily high dose of SMN-C1 from PND1, subjected to crush denervation between PND 31–41 and analyzed as above. Contralateral, non-denervated SOL from WT mice given a daily high dose of SMN-C1 also had a high fraction of endplates with short terminal sprouts (37.7 ± 6.3%, 34/90 total junctions), an statistically significant increased number of sprouts/endplate (0.60 ± 0.09; ANOVA: F (df 3, 446) = 6.302670; p = 0.0003; t value DMSO WT vs HD WT = 3.868508), and 27% of the sprouts (20/74 total sprouts) in these NMJs labeled positively for S100 but not for NF/SV2. These results suggest that these processes arose as consequence of drug treatment. Thus, SMN-C1 treatment appeared to induce short terminal sprouts in innervated endplates in WT and SMA mice.

### Crush-denervation induced nerve terminal sprouting in both WT and SMA mice

Imaging of crush-denervated SOL seven days after surgery showed increased sprouting was evident at many endplates in both WT and SMA muscles (Fig. 2B). In denervated DMSO WT SOL the % endplates with sprouts rose to 68.98 ± 8.00 (Fig. 3A, striped bars). In denervated LD SMA SOL it climbed to 44.52 ± 4.71% and in HD SMA SOL it reached 57.32 ± 6.90% (Fig. 3A, striped bars). Sprouts/endplate, which were all less than 1 in contralateral muscle, rose to close or well above 1 in denervated muscle for all groups (DMSO WT SOL: 1.34 ± 0.13; LD SMA SOL: 0.96 ± 0.10; HD SMA SOL: 1.16 ± 0.12) (Fig. 3B, striped bars). Sprout length, which averaged ~6 μm for the contralateral side (DMSO WT SOL: 7.43 μm ± 0.90; LD SMA SOL: 6.12 μm ± 1.26; HD SMA SOL: 7.06 μm ± 1.46), increased to over 25 μm on the denervated side (DMSO WT SOL: 26.25 μm ± 1.83; LD SMA SOL: 27.68 μm ± 1.96; HD SMA SOL: 29.52 μm ± 2.03) (Fig. 3C, striped bars). The changes in %endplates with sprouts, sprouts/endplate and sprout length in the denervated muscles were statistically significant relative to contralateral muscle in all treatment groups (t-test; Fig. 3). As expected, most sprouts in the denervated muscles stained positive for nerve and Schwann cell markers (Fig. 4C). Thus, reinnervation following a crush injury of the branch of the sciatic nerve that innervates the SOL induced similar increases in terminal sprouting measures in WT, LD and HD SMA mice.

### Reinnervation rate in pharmacological SMA mice was SMN-C1 dose-dependent

**Figure 5:**
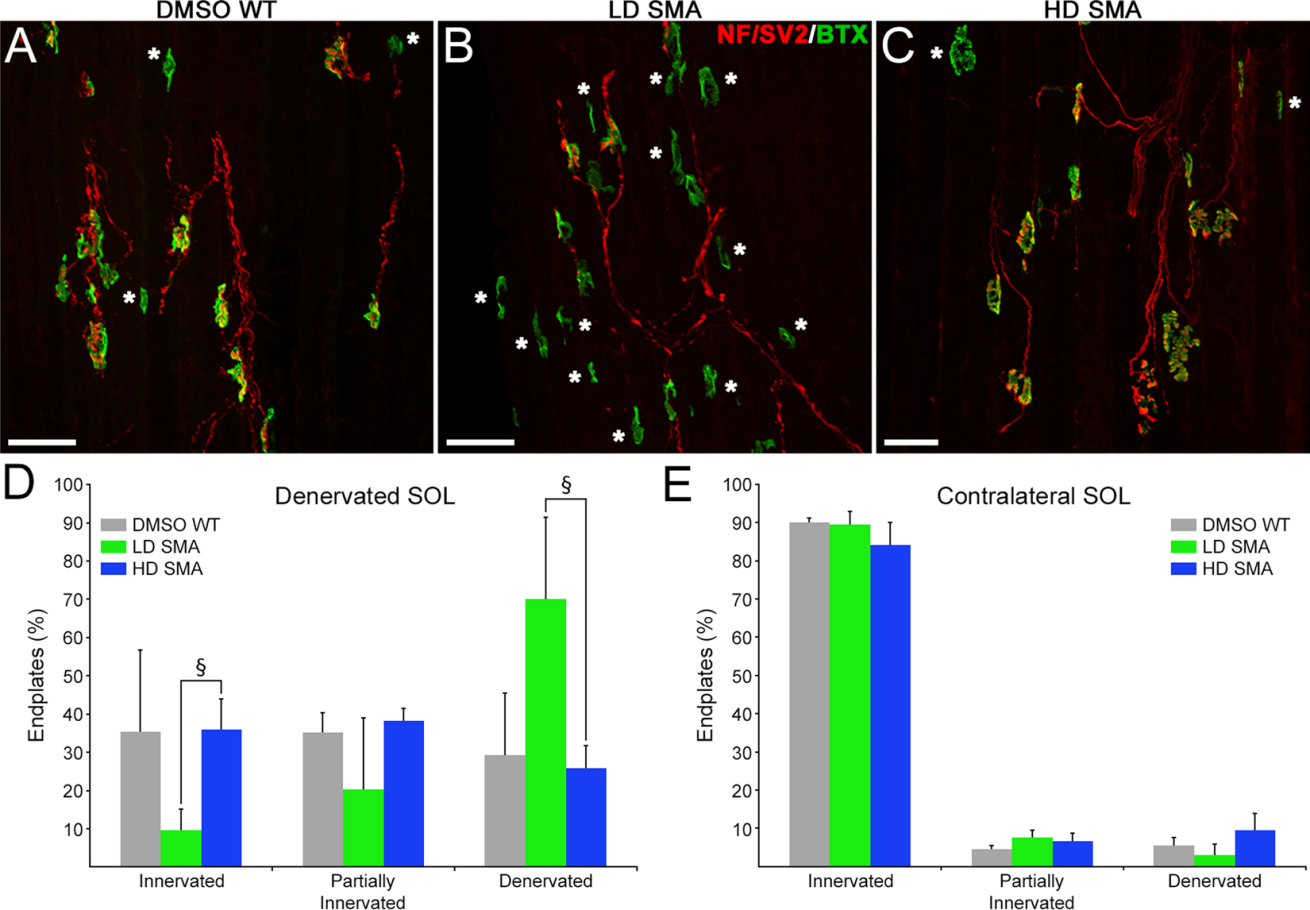
Slower reinnervation in LD SMA SOL was rescued in HD SMA SOL. (**A**–**C**) Representative fields from DMSO WT, LD SMA and HD SMA SOL longitudinal sections stained for AChR (green) and axons/nerve terminals (red), 7d after crush of the tibial branch of the sciatic nerve (Fig. 1). Denervated endplates are indicated by asterisks. Scale bar: 50 μm. **(D**) Quantification of innervation status on the denervated SOL. Sample sizes: DMSO WT: 2 mice, 144 endplates; LD SMA: 4 mice, 302 endplates; HD SMA: 4 mice, 355 endplates. ^§^p < 0.05, ANOVA with Bonferroni correction. (**E**) Quantification of innervation status on the contralateral SOL. Sample sizes: DMSO WT: 2 mice, 179 endplates; LD SMA: 4 mice, 312 endplates; HD SMA: 3 mice, 153 endplates. No significant differences were found in innervation status of contralateral muscles between treatment groups.

To assess reinnervation rate, SOL was denervated by crushing its nerve at the tibial nerve branch of the sciatic nerve, farther away from the muscle entry (Fig. 1, crush 2). One week later, muscles from operated and contralateral legs were dissected, frozen in liquid N_2_-cooled isopentane, and longitudinal sections were cut in a cryostat. Sections were stained for AChRs, axons and nerve terminals. For thick muscles like the SOL, antibody penetration is more efficient in sections than in whole-mount preparations, which allows a more reliable determination of innervation status. Endplates, both in operated and contralateral muscles, were scored as denervated, partially-innervated, or innervated if <~25%, ~25–75%, or >~75% AChR area was covered by the nerve, respectively. Innervation status on the contralateral, non-denervated muscle was similar between the three treatment groups with over ~90% endplates fully innervated (Fig. 5). However, on the muscles from the operated legs, LD SMA SOL denervated endplates were at ~70% (70.0% ± 21.4) and innervated endplates at ~10% (9.6% ± 5.6), while in HD SMA SOL denervated endplates were at ~25% (25.9% ± 6.0) and innervated ones were at 35% (35.9% ± 8.0), a comparable distribution to that found in DMSO WT SOL (denervated: 29.4% ± 16.2; innervated: 35.5% ± 21.3) (Fig. 5D). ANOVA with Bonferroni correction showed statistically significant differences between LD and HD SMA treatments on innervated junctions (ANOVA: F (df 2, 7) = 7.767; p = 0.0167; t value LD SMA vs HD SMA = 3.618) and denervated endplates (ANOVA: F (df 2, 7) = 8.937; p = 0.0118; t value LD SMA vs HD SMA = 3.956). Thus, the slower reinnervation rate of the LD SMA SOL seemed rescued in the HD SMA SOL.

### Terminal Schwann cells per NMJ area were slightly reduced in pharmacological SMA mice

**Figure 6:**
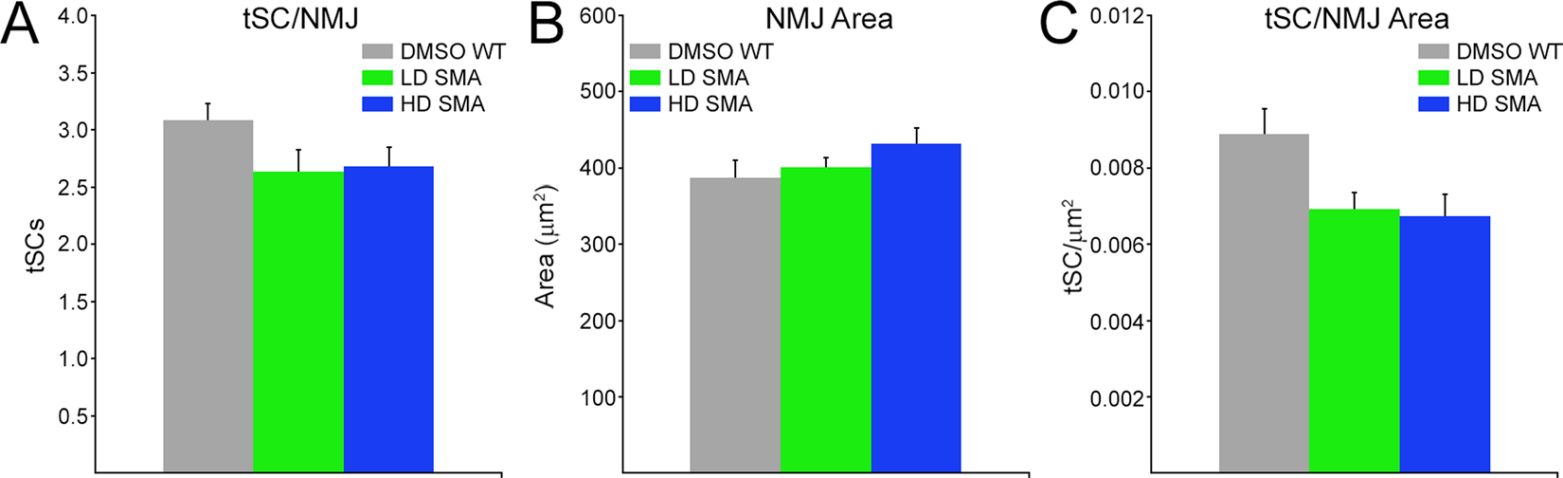
Slight but statistically significant reduction of tSCs/NMJ area in pharmacological SMA SOL. Number of tSCs per NMJ (**A**), NMJ AChR area (**B**) and tSC normalized to NMJ area (**C**) on non-denervated, contralateral muscles. Sample sizes: DMSO WT: 6 mice, 78 NMJs (**A**) 55 NMJs (**B**,**C**). LD SMA: 5 mice, 92 NMJs (**A**) 62 NMJs (**B,C**). HD SMA: 5 mice, 122 NMJs (**A**) 76 NMJs (**B**,**C**). ANOVA yielded p = 0.0301 for tSC/NMJ area but pairwise Bonferroni comparisons between groups were not significant.

The number of tSCs per NMJ, synaptic area, as measured by area of AChR staining, and tSCs per NMJ area, were quantified on PND31/32 contralateral SOL (Fig. 6). There were trends toward fewer tSCs per NMJ and larger synaptic area in LD and HD SMA SOL relative to WT DMSO control (Fig. 6A,B). None of these trends showed statistical significance by ANOVA. However, the downward trend in tSC number for both LD and HD SMA SOL was clearer when tSC number was normalized to NMJ area (Fig. 6C) and it reached statistical significance by ANOVA (tSCs/μm^2^, DMSO WT SOL: 0.0089 ± 0.0016; LD SMA SOL: 0.0069 ± 0.0009; HD SMA SOL: 0.0068 ± 0.0013. ANOVA: F(df 2, 13) = 4.643; p = 0.0301) (Fig. 6C). Bonferroni post hoc analysis yielded t values that approximated but failed to reach significance relative to DMSO WT control (t value DMSO vs LD SMA = 2.475; t value DMSO vs HD SMA = 2.708; t value LD SMA vs HD SMA = 0.224). This suggests that the reductions in tSCs/NMJ area between DMSO WT and SMA pharmacological mice were slight but statistically significant. This result also suggests that this parameter was insensitive to the SMN-C1 doses used.

## Discussion

Our data show that: (i) Short nerve terminal sprouts at non-denervated endplates were more abundant in SOL from SMN-C1 treated SMA mice than in DMSO WT controls. Treatment of WT mice with SMN-C1 suggested that these sprouts could be attributed to drug treatment and not to SMA phenotype. However, this cannot be unequivocally concluded until endplates from untreated SMAΔ7 mice are examined for the existence of these sprouts. (ii) Quantitative measures showed that pharmacological SMA mice are capable of terminal sprouting following reinnervation. (iii) Reinnervation was slower in LD SMA mice than in HD SMA mice or DMSO WT mice, which suggest that pharmacological SMA mice show defects in reinnervation upon nerve injury that can be rescued by optimal dosing of the *SMN2* splicing modifier.

SMN-C1 treatment induces a limited terminal sprouting response in non-denervated SOL endplates in WT and SMA mice. This conclusion was most clearly derived from (i) the statistically significant increase in sprouts/endplate in HD WT and HD SMA contralateral muscles and (ii) on the observation that these short sprouts (18–28% of the total) labeled only for S100 (Fig. 4) in the SMN-C1-treated muscles. None of these tSC sprouts were found in DMSO WT muscle. These data suggest that SMN-C1 may act on tSCs primarily to activate a sprouting response in these cells. Pharmacological SMA mice showed reduced tSCs/NMJ area relative to DMSO WT control (Fig. 6C). This finding might provide a less-likely, alternative explanation for the apparent “activation” of tSCs, at least in LD and HD SMA SOL. Thus, tSCs might be sprouting not because of the direct effects of SMN-C1 but to “compensate” for their having to cover a slightly larger NMJ area without an increase in their cell numbers (Fig. 6A,B). Regardless of mechanism, it is unclear whether the short sprouting induced by SMN-C1 at innervated endplates affects synaptic homeostasis. It appeared not to affect synaptic remodeling after reinnervation. Further work will be required to investigate if this short sprouting is observed in tSCs in other muscles.

Sprouting has been studied previously in various mouse models of SMA, representing different degrees of disease severity. Thus, selective neuronal deletion of the endogenous mouse SMN gene (*Smn*) led to a complete lack of terminal sprouting in the surviving young adults^20^. Because these animals completely lack SMN in neurons and supposedly harbor normal levels of SMN in other cells, they fail to properly model the human disease, where SMN, derived solely from *SMN2*, is reduced in all cells. Sprouting was also studied in the *Smn*^*2B*/−^ model^21^, in which disease onset is observed around PND 10 with an average lifespan of 28 days. Although terminal sprouting was elicited in this intermediate SMA model following reinnervation or muscle paralysis, it was less robust than in non-SMA mice. The axonal growth rate in *Smn*^*2B/*−^ mice was similar to that in non-SMA mice, and the reduced number of tSCs/NMJ in the former was proposed to be partly responsible for their impaired terminal sprouting response^21^. Compensatory nerve sprouting may be more relevant in milder forms of the human disease^22^. *Smn*^+/−^ mice, with a 50% reduction of SMN protein, are considered as a model of the mildest forms of SMA (type III/IV)^23^. Two studies have shown that nerves in *Smn*^+/−^ mice retain terminal sprouting capacity and that it accounts for their unchanged muscle force in the face of protracted motoneuron loss^18,19^. In terms of remaining SMN levels and average lifespan, the pharmacological mouse model used here (LD SMA mice) is comparable to the *Smn*^*2B*/−^ model. The capacity for terminal sprouting is largely preserved in both models. Unlike findings in the *Smn*^*2B*/−^ model^21^, we failed to detect a significant reduction in the number of tSCs/endplate in the LD SMA mice (Fig. 6). However, we did find a slight but statistically significant reduction in tSC number when it was normalized to synaptic area. This reduction was not sensitive to the dosage of SMN-C1 and had no impact on the terminal sprouting capacity of pharmacological SMA mice.

Reinnervation rate was slower in LD SMA than in DMSO WT or HD SMA mice (Fig. 5). This became evident when the denervation was done at the tibial branch, farther away from the muscle than at the soleus entry (Fig. 1). Analysis of contralateral muscles suggested that the innervation status prior to nerve crush was likely the same between HD SMA and LD SMA SOL (Fig. 5E). It remains to be determined whether the reduced rate of reinnervation in LD SMA mice is due to other reasons such as slower axon growth after injury, selective motoneuron-death induced by axon damage, or a general failure to thrive of the animals. It is important to note that this defect was rescued by raising SMN-C1 dose in the HD SMA mice (Fig. 5). In this context, reduction in axon growth was reported for motoneurons isolated from the severe *Smn*^−/−^*; SMN2*^+/+^ model mice^24^.

In summary, our data show that pharmacological SMA model mice are capable of terminal sprouting despite showing a slower rate of reinnervation. While terminal sprouting per se was marginally affected by the dose of the *SMN2* splicing modifier, reinnervation speed was critically dose-dependent. Further work is required to determine the cause of the reinnervation delay and the possible harnessing of the capacity to sprout for therapeutic purposes.

## Materials and Methods

### Animals

Care and treatment of all animals followed the National Institutes of Health Guide for the Care and Use of Laboratory Animals, and were approved by the Institutional Animal Care and Use Committees of the University of Southern California and Texas A&M University under animal use protocols 11136 and 2015-0353, respectively. SMN∆7 mice, and Wild-type mice (WT), non-SMA mice with the same genetic background were generated from breeder pairs of heterozygous SMN∆7 mice purchased from the Jackson labs (Strain #05025, FVB.Cg-Tg(SMN2*delta7)4299Ahmb Tg(SMN2)89Ahmb Smn1^*tm1Msd/J*^). Genotyping was performed as described previously^25^.

### Drug treatment

From postnatal day 1 (PND1) through the day before euthanasia (PND37/PND38 for all except 5 mice euthanized at PND46), homozygous SMNΔ7 mice were dosed daily via intraperitoneal injections with SMN-C1 (low dose (LD): 0.1 mg/kg or high dose (HD): 3 mg/kg, all as solutions in vehicle, 100% dimethyl sulfoxide (DMSO))^10^. Wild-type mice were dosed daily via the same route with DMSO. Some WT mice were also treated with HD SMN-C1 as above.

### Surgical procedures

Mice were anesthetized by inhalation with 1–3% isoflurane. Denervation of the right soleus (SOL) muscle was accomplished by crushing the soleus muscle nerve with #5 fine forceps either close to its entry into the muscle or at the tibial branch in the area of the popliteal fossa (Fig. 1).

### Immunostaining and confocal microscopy

Mice were euthanized by CO_2_ inhalation, and the denervated and contralateral SOL were dissected out. To study terminal sprouting, muscles were fixed in 4% paraformaldehyde (PFA) for 1 h and subjected to standard whole-mount immunofluorescence staining to label axons/nerve terminals with anti-neurofilament (NF) (2H3, Developmental Studies Hybridoma Bank), and anti-SV2 (Developmental Studies Hybridoma Bank), terminal Schwann cells (tSCs) with anti-S100 (Dako), and acetylcholine receptors (AChRs) with α-bungarotoxin (BTX, Life Technologies). To study innervation status, muscles were fixed overnight in 4% PFA, cryoprotected in sucrose overnight, and sliced in a HM 550 cryostat (Microm) into 40 μm-think longitudinal sections. Sections were subjected to immunostaining to label axons/nerve terminals and AChRs as above. Anti-synaptophysin (Life Technologies) was used occasionally to label nerve terminals in longitudinal sections. All preparations were imaged on a F10-ASW Fluoview confocal microscope (Olympus), with 20X (numerical aperture (NA) 0.85) and 60X (NA 1.35) oil immersion objectives.

### Quantification of terminal sprouting, innervation status and tSC numbers

Sprout analysis was restricted to endplates on bundles of surface fibers to avoid studying endplates that failed to be stained by lack of penetration of antibodies. Sprouting was assessed on confocal stacks, built using Metamorph software. Each endplate in the stack, regardless of its innervation status, was analyzed for presence and number of nerve (NF+/SV2+) or tSC (S100+) sprouts and nerve or tSC sprout length (μm). Sprouts were tracked through the optical section to ensure that they were terminal sprouts. For the %endplates with sprouts parameter, data were recorded per muscle but averaged and presented per treatment group. For the sprouts/ endplate and sprout length parameters, data were pooled and averaged per treatment group.

Innervation status was quantified on endplates from longitudinal sections that were scored as denervated, partially-innervated, or innervated if <~25%, ~25–75%, or >~75% AChR area was covered by the nerve, respectively. Data were recorded per muscle, averaged and presented per treatment group.

Number of tSCs and endplate area (μm^2^), defined as the area stained for BTX, were quantified on “en-face” endplates from contralateral, non-denervated muscles only. Data were averaged per muscle and presented per treatment group.

### Statistical analysis

Number of animals, endplates and sprouts analyzed are indicated in the Figure legends. Numerical values are expressed either as percentages of the total or as mean ± SEM. Two-sample, two-tailed Student t tests and analysis of variance (ANOVA) with Bonferroni multiple comparison test were used to compare means. Prism 5 (Graphpad) and Excel (Microsoft) were used to compute statistics. Significance was set at P values of < 0.05 for * (t test) or § (ANOVA) and of <0.01 for ** (t test) or §§ (ANOVA).

## Data Availability

The data that support this study are available from the corresponding authors upon reasonable request.
